# Cigarette Smoking Impairs Cardiorespiratory and Metabolic Response at Peak Incremental Exercise and during Recovery in Young, Physically Active Adults

**DOI:** 10.1249/MSS.0000000000003602

**Published:** 2024-11-12

**Authors:** MARTA BORRELLI, CHRISTIAN DORIA, NICHOLAS TONINELLI, STEFANO LONGO, GIUSEPPE CORATELLA, EMILIANO CÈ, SUSANNA RAMPICHINI, FABIO ESPOSITO

**Affiliations:** Department of Biomedical Sciences for Health, Università degli Studi di Milano, ITALY

**Keywords:** SMOKER, HEART RATE, KINETICS, PEAK EXERCISE, PULMONARY OXYGEN UPTAKE, RECOVERY

## Abstract

**Purpose:**

Cigarette smoking (CS) induces systemic changes that impair cardiorespiratory and muscular function both at rest and during exercise. Although these abnormalities are reported in sedentary, middle-aged smokers (SM) with pulmonary disease, few and controversial studies focused on young, physically active SM at the early stage of smoking history. This study aimed at assessing the effect of CS on cardiorespiratory and metabolic response during an incremental test and the subsequent recovery in young, physically active SM without known lung or cardiovascular disease.

**Methods:**

After pulmonary function evaluation, 12 SM (age: 22±2 yr; body mass: 75±8 kg; stature: 1.78±0.06 m; 12±4 cigarette per day for 6±2 yr; mean ± SD) and 12 non-SM (control group; age: 23±1 yr; body mass: 76±8 kg; stature: 1.79±0.08 m) matched for age and exercise habits underwent an exhaustive incremental step test (25 W/2 min) on a cycle ergometer. Pulmonary O_2_ uptake (V̇O_2_), expiratory ventilation (V̇_E_), heart rate (*f*_H_) responses and lactate concentration were assessed during the test and subsequent recovery.

**Results:**

Despite similar static lung volumes, SM reported lower peak expiratory flow (−23%; *P* = 0.003) and maximal voluntary ventilation (−10%; *P* = 0.003). At submaximal exercise, no differences in the cardiorespiratory and metabolic were noted between the two groups. However, SM exhibited ventilatory (*P* < 0.01) and lactate thresholds at lower work rates (*P* = 0.01). At peak exercise, SM exhibited lower V̇O_2_ (−8%; *P* = 0.02), mechanical power (−11%; *P* = 0.02), and V̇_E_ (−9%; *P* = 0.01). During recovery, SM showed longer time constants (τ) in V̇O_2_ (+52%; *P* = 0.002), V̇_E_ (+19%; *P* = 0.027) and *f*_H_ (+21%; *P* = 0.022) and smaller *f*_H_ at 30 s of recovery (HRR30; −31%; *P* = 0.032).

**Conclusions:**

These results are compatible with an early CS-related impairment of the cardiorespiratory and metabolic function even in young individuals with relatively short smoking history.

Cigarette smoking (CS) is one of the most impactful risk factors for cardiovascular morbidity and mortality, representing the primary preventable cause of death worldwide (1). The cigarette compounds, indeed, have detrimental effects on various apparatus, among which the cardiorespiratory and muscular systems (2–4). Carbon monoxide (CO) bound to hemoglobin (HbCO) impairs the O_2_ delivery (4,5); tar, produced by tobacco combustion, increases pulmonary airway resistance and alveolar–capillary barrier thickness (5,6) and, in turn, the work of breathing (5,7); nicotine, stimulating the sympathetic nervous system, increases resting heart rate (*f*_H_), cardiac work (8,9), and peripheral vasoconstriction (10). Lastly, cigarettes contain reactive oxygen species and other oxidants that lead to a decline in mitochondrial function and to skeletal muscle dysfunction (11).

These detrimental effects have been reported to affect the cardiorespiratory, muscular, and metabolic response to exercise, resulting in exercise intolerance (4). Indeed, reduction in exercise capacity has been reported during submaximal exercise, resulting in higher *f*_H_ (7,9,12), reduced gas exchange (i.e., increase in ventilatory equivalent for oxygen, V̇_E_/V̇_O2_ and for carbon dioxide, V̇_E_/V̇CO_2_), and increased blood lactate concentration ([La^−^]) at the same work rate (7,8,13).

Extensive evidence demonstrated the association between CS and reduced maximum pulmonary oxygen uptake (V̇O_2max_) and *f*_H max_ in sedentary, middle-aged smokers (SM) with (13–15) and without chronic obstructive pulmonary disease (COPD) (16,17).

Poor consideration has been given so far to young, physically active SM at the early stage of smoking history without known lung or cardiovascular disease, with controversial results (8,12,18–23). Indeed, in some studies, the control group (CTRL) was not present (12,18,19), some failed to match SM to CTRL for exercise habits (8,20,21), and some others focused only on lung function evaluation (22,23). Therefore, limited attention has been therefore paid to the evaluation of the possible effect of CS on cardiorespiratory and metabolic responses not only to physical exercise but also to the subsequent recovery phase. To this purpose, the measurement of heart rate recovery (HRR), which refers to the rate at which the *f*_H_ returns to baseline after physical exertion (8,24,25), may give useful information. Indeed, after exercise cessation, the autonomic nervous system responds with a prompt reactivation of the vagal and gradual withdrawal of the sympathetic tone, decreasing *f*_H_ to baseline levels. Consequently, the rate of *f*_H_ decrease has been widely acknowledged as a powerful marker of autonomic dysfunction and of the overall cardiorespiratory fitness and a predictor of all-cause mortality in both asymptomatic and diseased populations (24,26–29). During the recovery phase, also the V̇O_2_ kinetics, which seems to be a more reliable indicator of cardiovascular dysfunction compared with the on-kinetics (30), can provide insights on the possible degree of impairment of the cardiorespiratory and metabolic systems (31). Very few studies analyzed the V̇O_2_ recovery in SM with COPD, reporting delays in cardiorespiratory and metabolic kinetics (32,33). The underlying physiological mechanisms, though, were not deeply investigated.

Therefore, the aim of this study was to determine the effect of CS on the cardiorespiratory and metabolic response to a maximal incremental test and during the recovery among young, physically active SM without known lung or cardiovascular disease. We hypothesized that SM would present (i) an exaggerated cardiorespiratory and metabolic response to a stepwise incremental exercise at the same work rate and (ii) delayed recovery kinetics of the cardiorespiratory and metabolic variables compared with non-SM. Indeed, despite their relatively young age, short smoking history, and high level of physical fitness, which may mitigate partially the CS detrimental effects, SM of the present investigation may have already developed alterations in oxidative stress, inflammatory state, autonomic response, and O_2_ delivery that could alter the physiological response to exercise and the subsequent recovery phase.

## METHODS

### Participants

Based on pilot findings, a large Cohen’s *d* effect size (1.2) in the differences of the time constant (*τ*) of the cardiorespiratory response during recovery between SM and CTRL was used for a two-tailed unpaired Student’s *t*-test to calculate the optimal sample size (G-Power 3.1, Dusseldorf, Germany). Considering a required power (1 − β) = 0.80 and an *α* = 0.05, the desired sample size resulted in 24 participants.

Thereafter, 24 young, physically active males (12 SM and 12 CTRL) were recruited. CTRL was matched for age and exercise habits (International Physical Activity Questionnaire [IPAQ]; 4304 ± 1675 vs 4107 ± 1608 METs; for CTRL and SM, respectively; *P* = 0.772). The anthropometric characteristics are given in Table 1. The inclusion criteria for CS were smoking at least 6 cigarettes per day and a history of CS for a minimum of 2 continuous years (34). The exclusion criteria for both groups were as follows: (i) cardiovascular and respiratory diseases, (ii) musculoskeletal impairments, and (iii) medications altering cardiovascular and respiratory responses.

**TABLE 1:** : Anthropometric and demographic characteristics of SM and CTRL expressed as mean ± SD.

	CTRL, *n* = 12	SM, *n* = 12
Age (yr)	22.8 ± 1.4	21.6 ± 2.2
Body mass (kg)	76.3 ± 8.1	75.2 ± 8.1
Stature (m)	1.79 ± 0.08	1.7 ± 0.06
Cigarettes per day (*n*)	–	12 ± 4
History of smoking (yr)	–	6 ± 2

Participants were fully informed about the study’s purpose and the experimental design and gave written consent to participate. The study was approved by the ethics committee of the local university (no. 77/20) and was conducted in accordance with the latest principles of the Declaration of Helsinki.

### Experimental Design

Participants visited the laboratory twice, with at least 48 h in between. During the first session, after becoming familiar with the experimental procedures and completing anthropometric assessments, participants underwent pulmonary function evaluations. On the second session, they performed an incremental test to determine the V̇O_2max_, the maximal mechanical aerobic power ( ${\dot{W}}_{\max}$), and the cardiorespiratory and metabolic kinetics during the recovery phase.

### Experimental Procedures

All tests were conducted in a climate-controlled laboratory (constant temperature of 20 ± 1°C and relative humidity of 50 ± 5%) at approximately the same time of the day to minimize any possible bias caused by circadian rhythms. Prior to each testing day, participants were instructed to abstain from caffeine and any other stimulant substances for at least 12 h, as well as refrain from heavy exercise for at least 24 h. SM were asked to smoke their last cigarette 1.5 h before testing to allow 5–16% elimination of blood HbCO levels in order to avoid the acute effects of CS (5).

#### Familiarization, anthropometric, and pulmonary function assessment

During the first session, the participants were equipped with a face mask and wearable devices to familiarize with the equipment used for cardiopulmonary testing. For each participant, optimal saddle height, handlebar angle of inclination, and pedal foot-belt position were defined (35). Body mass and stature were measured to the nearest 0.1 kg and 1 cm, respectively, by a mechanical scale equipped with a stadiometer (Asimed, Samadell, Barcellona).

On the same day, participants underwent a spirometric evaluation to determine vital capacity (VC) and dynamic lung volumes (forced vital capacity [FVC]; forced expired volume in the first second of the test [FEV_1_]; forced expiratory volume during the sixth second of the test [FEV_6_]; forced expiratory flow at 25% and 75% of the pulmonary volume [FEF 25–75%]; instantaneous expiratory flow when 25% of FVC has to be expired [MEF75%]; instantaneous expiratory flow when 50% of FVC has to be expired [MEF50%]; instantaneous expiratory flow when 75% of FVC has to be expired [MEF25%]; forced expiratory time [FET 100%]; peak expiratory flow [PEF]; expiratory reserve volume [VRE]; inspiratory reserve volume [IRV]; and maximal voluntary ventilation [MVV]). Maximal inspiratory and expiratory pressure (MIP and MEP, respectively) were measured at the mouth using a portable manometer equipped with a mouthpiece (S&M Instrument Company Inc., mod. PortaResp, Doylestown, PA). Predicted values were determined according to Miller et al. (36). After familiarization with the manometer, participants repeated the maneuver three times, and the highest value was considered.

#### Incremental exercise test

On the second visit, V̇O_2max_ and ${\dot{W}}_{\max}$ were determined by a stepwise incremental test (37). After three minutes of baseline recordings and four minutes of 100 W warm-up, work rate was raised by 25 W every two minutes until task failure. Participants were instructed to maintain the pedaling rate between 60 and 70 rpm. Task failure was defined as the inability to maintain the cadence within the 5 rpm of the imposed range for more than 5 s (38). After exercise cessation, a five-minute of active recovery at 30 W at 30 rpm was included.

[La^−^] was measured at rest, at the end of each work rate and at minutes 1, 3, and 5 of recovery to assess the [La^−^] at peak. At the same time, participants were asked to indicate their rate of perceived exertion (RPE) on a general (RPE_GEN_; Borg 6–20), muscular, and respiratory (RPE_MUSC_ and RPE_RESP_, respectively; CR-10) standpoints.

### Measurements

Tests were performed on an electromechanically braked cycle ergometer (mod. 839E, Monark, Sweden). During the experiments, work rate and cadence were continuously recorded. Expiratory ventilation (V̇_E_), V̇O_2_, respiratory frequency (*f*_R_), tidal volume (V_T_), and carbon dioxide production (V̇CO_2_) were measured on a breath-by-breath basis by a metabolic unit consisting of a turbine flowmeter, a zirconium oxygen sensor, and an infrared CO_2_ meter (Quark b^2^, Cosmed, Rome, Italy). Moreover, V̇_E_/V̇_O2_ and V̇_E_/V̇CO_2_, end-tidal oxygen pressure (PetO_2_), end-tidal carbon dioxide pressure (PetCO_2_), and respiratory exchange ratio (RER) were calculated. According to manufacturer’s instructions, turbine and gas analyzers were calibrated before each test with a 3-L syringe (mod. 5530, Hans-Rudolph, Shawnee, KS) and a certified gas mixture of known concentration (16% O_2_, 5% CO_2_, balance N_2_). *f*_H_, stroke volume (SV), and cardiac output ( $\dot{Q}$) were acquired with PhysioFlow® Imped monitor (Manatec Biomedical, Paris, France). Lastly, 20 μL arterialized blood samples were collected from the ear lobe and analyzed by an enzymatic–amperometric system (Labtrend, Bio Sensor Technology GmbH, Berlin, Germany) to determine [La^−^].

### Data Analysis

All data were analyzed offline. Respiratory and gas exchange responses were edited of spurious breaths that resulted from swallowing, coughing, sighing, or premature ending of breath, by deleting values outside three SD from the local mean (39).

V̇O_2max_ was defined as the value obtained from the plateau in the relationship between V̇_O2_ and $\dot{W}$ during the incremental stepwise test. In the event that the plateau did not occur, the highest value of V̇O_2_ was considered as the maximum if (i) *f*_H_ did not increase between consecutive loads and (ii) RER > 1.1 (37). The ${\dot{W}}_{\max}$ was determined as the mechanical work rate corresponding to the intersection between the V̇O_2_ plateau and the linear relationship between V̇O_2_ and $\dot{W}$. The first ventilatory threshold (VT_1_) was defined as V̇O_2_ at which V̇_E_/V̇O_2_ and Pet_O__2_ increased with time with no concomitant increase in V̇_E_/V̇CO_2_ and decrease in PetCO_2_ (40). The second ventilatory threshold (VT_2_) was calculated as the V̇_O2_ corresponding to the work rate at which V̇_E_/V̇_O2_ and V̇_E_ showed a second nonlinear increase with time, whereas V̇_E_/V̇CO_2_ increased and PetCO_2_ began to fall (40). V̇O_2_ values corresponding to the ventilatory thresholds were reported as the average of the values detected by three experienced independent operators. The compensated area of incremental stepwise exercise, which reflects the blood buffering systems intervention, was calculated as the difference between VT_1_ and VT_2_ (41).

The lactate accumulation point (LT) was determined by the DMAX method, which identifies the point on the third-order polynomial curve that results in the maximum perpendicular distance from the straight line connecting the two outermost data points (42).

For the kinetic analysis, the data of the recovery phase of all cardiorespiratory and metabolic variables were linearly interpolated to 1-s intervals and fitted by monoexponential of this form:


$$Y\left(t\right)=Y_{0}+\mathrm{AMP}\;\left[1-e^{-\frac{\left(t-t_{D}\right)}{\tau }}\right],$$

where *Y*_0_ constitutes the value of the cardiorespiratory variables at the end of exercise, AMP is the amplitude of the response during recovery, *τ* is the time necessary to reach the 63% of the response, and *t*_D_ is time delay of the exponential function. The model parameters were estimated by least-squares nonlinear regression (Origin, OriginLab Corp., Northampton, MA) in which the best fit was determined by minimization of the residual sum of squares. The monoexponential model was applied considering that the recovery kinetics are less influenced by the cardiodynamic phase, which requires the double-exponential model (30,43).

The *f*_H_ recovery response is characterized by a rapid decay immediately after exercise cessation (i.e., fast phase), attributable mainly to parasympathetic reactivation and by more gradual decay (i.e., slow phase) until the return to baseline values, due to parasympathetic reactivation and sympathetic withdrawal (25,44). As HRR indexes of the fast phase, HRR30s and HRR60s were calculated as the differences between peak *f*_H_ at the end of exercise and the *f*_H_ value reached after 30 s and 1 min of recovery, respectively (26). T30 is an additional index of the fast phase. It refers to the negative reciprocal of the slope of the regression line (−1/slope) of the logarithmically transformed *f*_H_ during the first 30 s (26,44). A lower T30 value indicates greater parasympathetic activity. The slow phase of the *f*_H_ recovery is described by *τ* of the exponential function fitting the *f*_H_ recovery phase.

### Statistical Analysis

Descriptive statistics were used to define the study sample characteristics. The Shapiro–Wilk test was applied to check the normal distribution. A two-way repeated-measures ANOVA checked for differences between groups over work rate during the stepwise incremental test. For all pairwise multiple comparisons, the Bonferroni’s correction was applied. The differences between the two groups of cardiorespiratory and metabolic parameters were detected by one-tailed unpaired Student’s *t*-test (one-tailed was chosen because we expected that SM exhibit lower cardiopulmonary value and higher *τ*). The Hedge’s *g* effect size with 95% confidence interval (95% CI) was also calculated and interpreted as follows: trivial, 0.00–0.19; small, 0.20–0.59; moderate, 0.60–1.19; large, 1.20–1.99; very large, ≥2.00 (45). All statistical analyses were performed by using statistical software (IBM SPSS Statistics v. 28, Armonk, NY). The significance level was set at *α* < 0.05. Results are presented as mean ± SD.

## RESULTS

Table 2 reports the pulmonary function outcomes in the two groups.

**TABLE 2:** Respiratory function test parameters in SM and CTRL.

	CTRL			SM	
	Absolute	Predicted	% Predicted	Absolute	Predicted	% Predicted
FVC (L)	5.8 ± 0.7	5.4 ± 0.5	108 ± 7	5.6 ± 0.7	5.4 ± 0.4	105 ± 12
FEV_1_ (L)	5.0 ± 0.6	4.6 ± 0.4	109 ± 9	4.7 ± 0.5	4.6 ±0.3	103 ± 10
FEV_6_ (L)	5.8 ± 0.7	5.5 ± 0.6	105 ± 7	5.5 ± 0.7	5.6 ± 0.4	99 ± 10
FEV_1_/FVC	85 ± 4	83 ± 0.4	103 ± 5	83 ± 9	83 ± 0.5	100 ± 10
FEF 25–75%	5.4 ± 1.1	5.2 ± 0.2	104 ± 21	4.9 ± 1.2	5.2 ± 0.1	94 ± 22
MEF75%	8.9 ± 1.6	8.7 ± 0.5	102 ± 16	7.4 ± 1.8*	8.6 ± 0.4	86 ± 21*
MEF50%	6.0 ± 1.6	5.7 ± 0.3	104 ± 27	5.5 ± 1.3	5.7 ± 0.2	96 ± 23
MEF25%	3.0 ± 0.8	2.7 ± 0.2	110 ± 32	2.7 ± 0.7	2.8 ± 0.2	100 ± 28
FET 100%	5.4 ± 2.2	–	–	7.7 ± 3.6	–	–
PEF (L·s^−1^)	10.3 ± 1.9	10.2 ± 0.5	101 ± 16	8.0 ± 1.9*	10.2 ± 0.4	78 ± 18*
VRE (L)	1.8 ± 0.5	1.7 ± 0.1	108 ± 30	1.9 ± 0.4	1.7 ± 0.1	113 ± 26
IRV (L)	2.6 ± 0.6	–	–	2.2 ± 0.7	–	–
MVV (L·min^−1^)	192 ± 22	155 ± 11	123 ± 8	172 ± 16*	157 ± 8	110 ± 12*
MIP (cmH_2_O)	117 ± 14	112 ± 1	102 ± 17	115 ± 20	112 ± 1	103 ± 18
MEP (cmH_2_O)	123 ± 28	147 ± 1	84 ± 19	124 ± 15	148 ± 1	84 ± 10

Data are presented as mean ± SD. The predicted equations were obtained according to Miller et al. (36).

* *P* < 0.05 vs CTRL.

FVC, forced vital capacity; FEV_1_, forced expiratory volume during the first second of the test; FEV_6_, forced expiratory volume during the sixth second of the test; FEF 25–75%, forced expiratory flow at 25% and 75% of the pulmonary volume; MEF75%; instantaneous expiratory flow when 25% of FVC has to be expired; MEF50%, instantaneous expiratory flow when 50% of FVC has to be expired; MEF25%, instantaneous expiratory flow when 75% of FVC has to be expired; FET 100%, forced expiratory time; PEF, peak expiratory flow; VRE, expiratory reserve volume; IRV, inspiratory reserve volume; MVV, maximal voluntary ventilation; MIP, maximal inspiratory mouth pressure; MEP, maximal expiratory mouth pressure.

**FIGURE 1:**
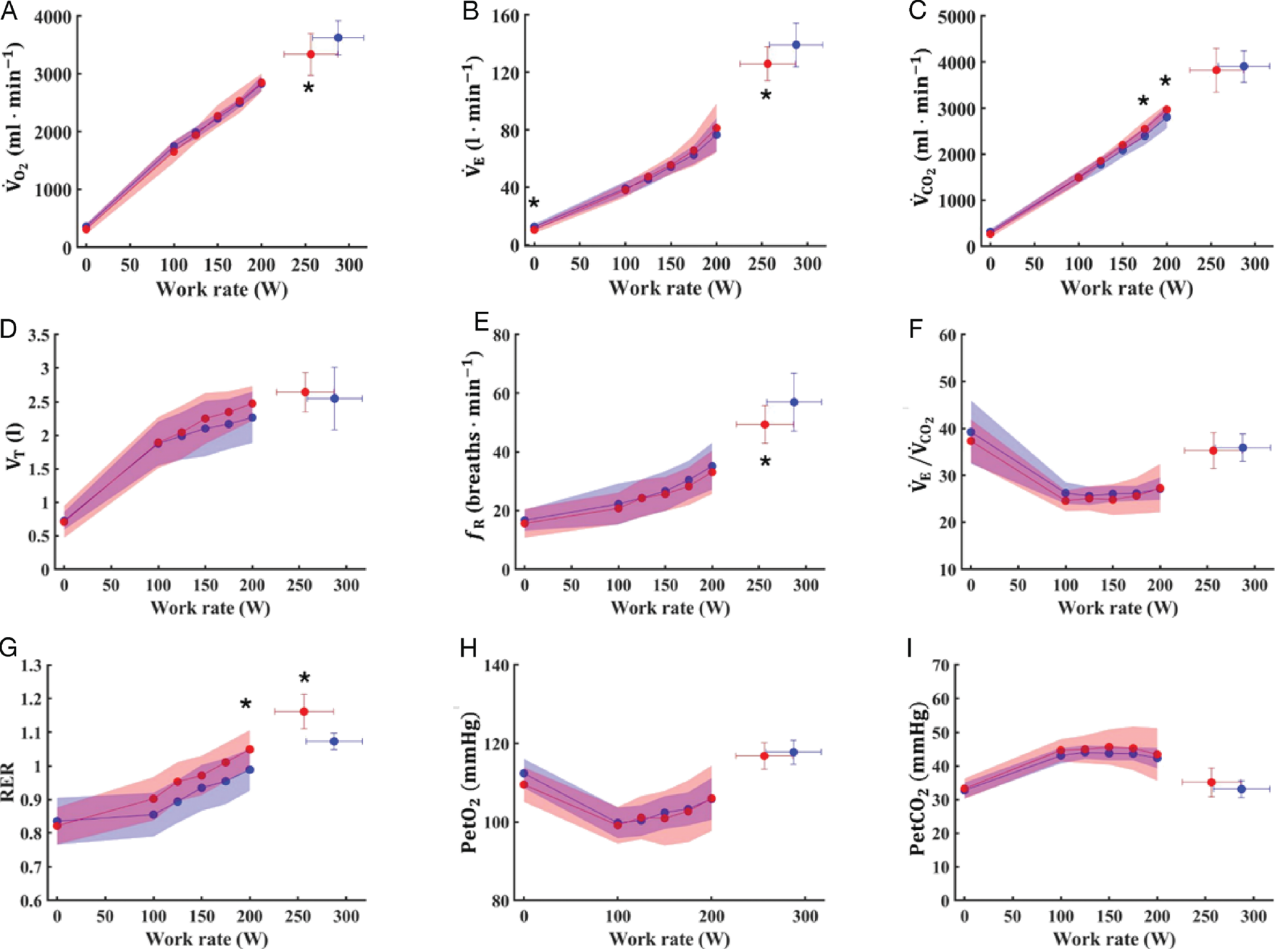
Ventilatory and metabolic responses to incremental exercise in smokers (*red circles*) and controls (*blue circles*). V̇O_2_, pulmonary oxygen uptake (A); V̇_E_, minute ventilation (B); V̇CO_2_, carbon dioxide production (C); V_T_, tidal volume (D); *f*_R_, respiratory frequency (E); V̇_E_/V̇C_O2_, ventilatory equivalent for carbon dioxide (F); RER, respiratory exchange ratio (G); PetO_2_ end-tidal oxygen pressure (H); PetCO_2_, end-tidal carbon dioxide pressure (I). Data are presented as mean ± SD; * *P* < 0.05 vs CTRL.

**FIGURE 2:**
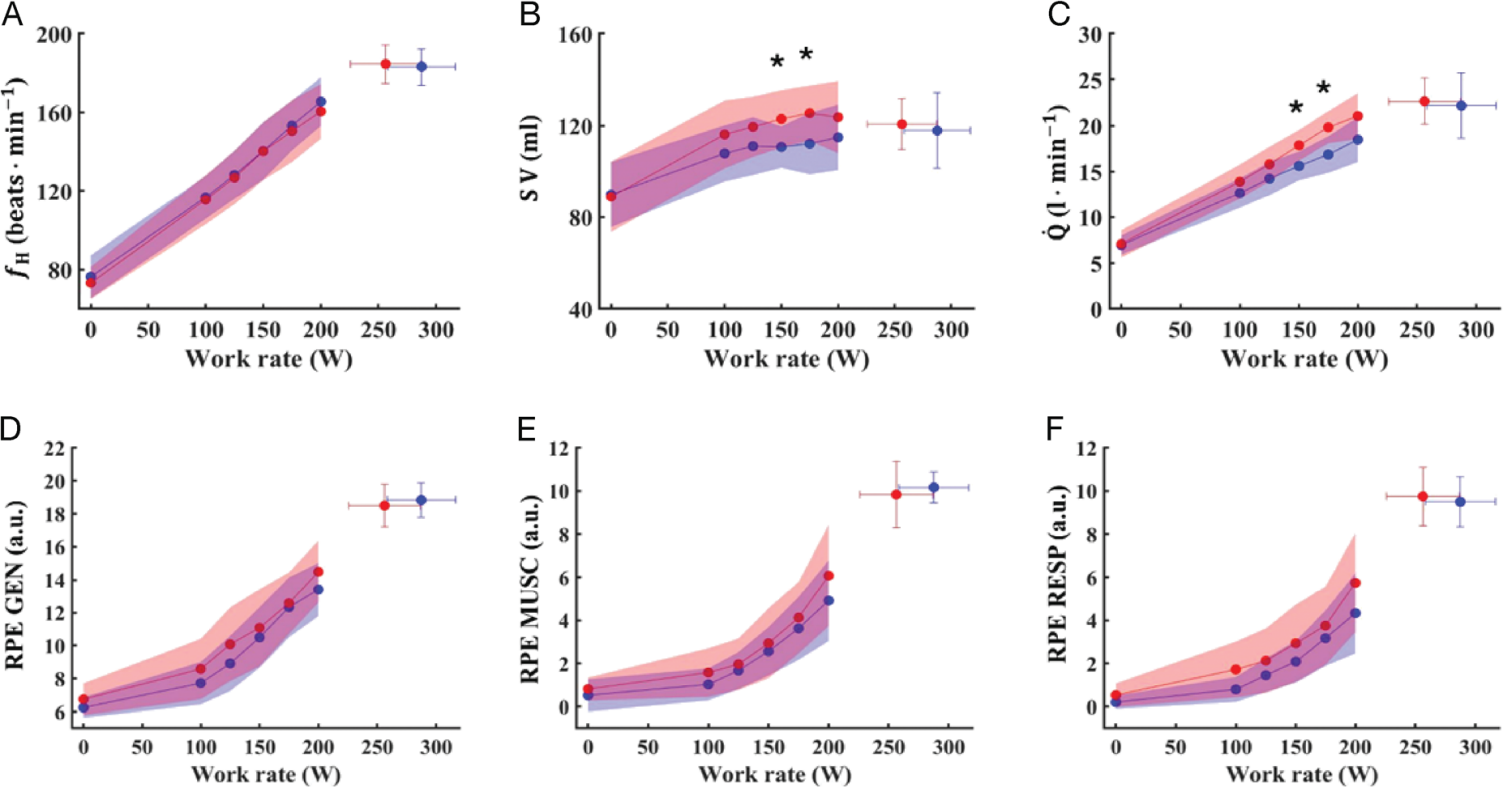
Cardiovascular and perceived exertion responses to incremental exercise in smokers (*red circles*) and controls (*blue circles*). *f*_H_, heart rate (A); SV, stroke volume (B); $\dot{Q}$, cardiac output (C); RPE General, rate of general perceived exertion (D); RPE Muscular, rate of muscular perceived exertion (E); RPE Respiratory, rate of respiratory perceived exertion (F). Data are presented as mean ± SD; * *P* < 0.05 vs CTRL.

**FIGURE 3:**
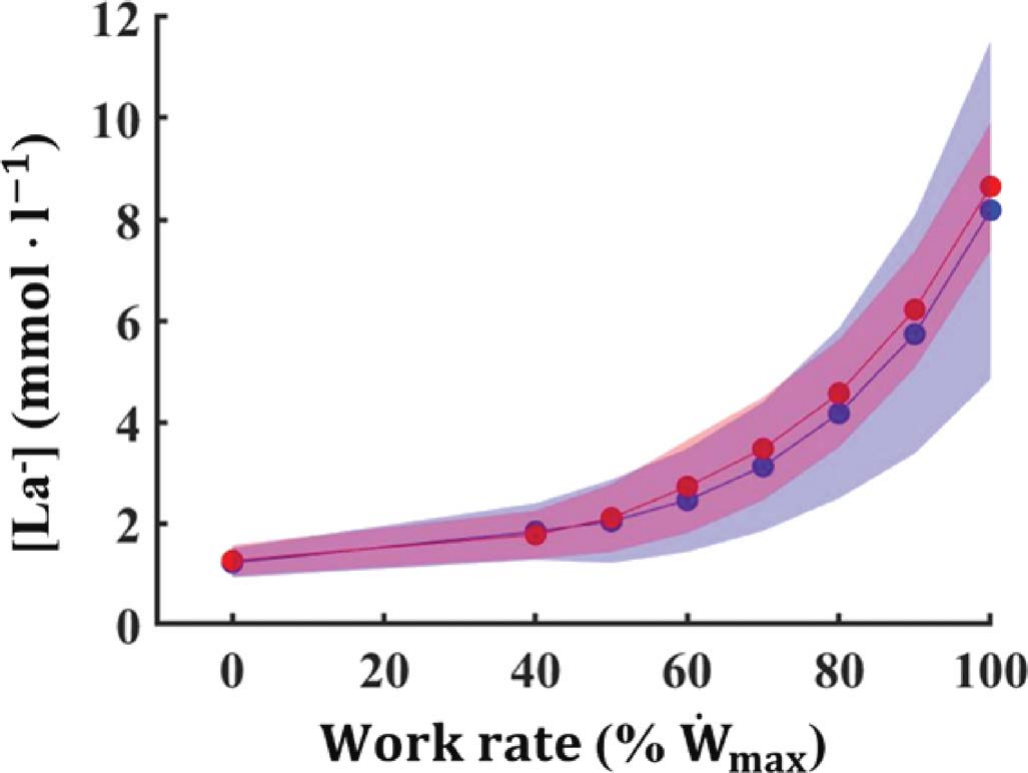
[La^−^], blood lactate concentration during the incremental cycle exercise in smokers (*red circles*) and controls (*blue circles*). Data are presented as mean ± SD; * *P* < 0.05 vs CTRL.

Figure 1, Figure 2, and Figure 3 show the respiratory, metabolic, and cardiovascular responses to the incremental test in the two groups. No differences were noted between the two groups in most of the cardiorespiratory and metabolic parameters during baseline recordings. Indeed, similar value between SM and CTRL were found for *f*_H_ (77 ± 12 vs 73 ± 8 beats·min^−1^, respectively), V̇O_2_ (309 ± 87 vs 353 ± 48 mL·min^−1^, respectively), V̇CO_2_ (262 ± 76. vs 308 ± 53 mL·min^−1^, respectively), *f*_R_ (17 ± 4 vs 19 ± 5 breaths·min^−1^, respectively), SV (89 ± 15 vs 90 ± 14 mL, respectively), and $\dot{Q}$ (7.1 ± 1.5 vs 6.9 ± 1.0 L·min^−1^, respectively), but SM had lower V̇_E_ compared with CTRL (10.3 ± 2.5 vs 12.3 ± 2.0 L·min^−1^, respectively; *P* = 0.024; *g* = −0.83, moderate; 95% CI = −0.01–1.66).

Despite similar *f*_H max_ (184 ± 8 vs 184 ± 9 beats·min^−1^ for SM and CTRL, respectively; Fig. 2A), SM reached lower V̇O_2max_ (3334 ± 362 vs 3625 ± 295 mL·min^−1^ for SM and CTRL, respectively; *P* = 0.022; *g* = −0.84, moderate; 95% CI = 0.01–1.67; Fig. 1A) and ${\dot{W}}_{\max}$ (256 ± 30 vs 288 ± 29 W, respectively; *P* = 0.009; *g* = −1.01, moderate; 95% CI = 0.16–1.86) compared with CTRL. Moreover, SM exhibited lower V̇_E max_ (126 ± 12 vs 139 ± 15 L·min^−1^ for SM and CTRL, respectively; *P* = 0.013; *g* = −0.94, moderate; 95% CI = 0.10–1.79; Fig. 1B) and *f*_R max_ (49 ± 6 vs 57 ± 9 breaths·min^−1^ for SM and CTRL, respectively; *P* = 0.011; *g* = −0.97, moderate; 95% CI = 0.12–1.81; Fig. 1E) compared with CTRL. Conversely, no differences were noted in V_T max_ (2.6 ± 0.4 vs 2.5 ± 0.4 L for SM and CTRL, respectively; Fig. 1D) and [La^−^] at peak (9.2 ± 1.9 vs 9.4 ± 1.9 mmol·L^−1^ for SM and CTRL, respectively; Fig. 3). No differences were observed between SM and CTRL in RPE_GEN_, RPE_MUSC_, and RPE_RESP_ (Fig. 2D, E, and F, respectively).

At VT_1_, SM showed lower work rate (180 ± 18 vs 202 ± 26 W, respectively; *P* = 0.015; *g* = −0.92, moderate; 95% CI = 0.08–1.76) and V̇O_2_ (2589 ± 324 vs 2816 ± 320 mL·min^−1^ for SM and CTRL, respectively; *P* = 0.050; *g* = −0.68, moderate; 95% CI = −0.14–1.50) compared with CTRL. Similarly, at VT_2_, SM reached lower work rate (219 ± 24 vs 251 ± 31 W, respectively; *P* = 0.005; *g* = −1.12, moderate; 95% CI = 0.26–1.98) and V̇_O2_ and at VT_2_ (3024 ± 394 vs 3352 ± 382 mL·min^−1^ for SM and CTRL, respectively; *P* = 0.025; *g* = −0.82, moderate; 95% CI = −0.02–1.65). However, these differences disappeared when data were expressed as percentage of V̇O_2max_ for both VT_1_ (77.5 ± 2.8 vs 77.6 ± 4.8%, for SM and CTRL, respectively) and VT_2_ (90.5 ± 5.1 vs 92.3 ± 5.1% V̇O_2max_ for SM and CTRL, respectively). Similarly, no differences were found between groups when VT_1_ and VT_2_ were expressed as percentage of ${\dot{W}}_{\max}$ (VT_1_: 73.8 ± 3.2 vs 72.3 ± 5.2 % ${\dot{W}}_{\max}$, for SM and CTRL, respectively; and VT_2_: 89.9 ± 4.5% vs 90.9 ± 5.4% ${\dot{W}}_{\max}$ for SM and CTRL, respectively). The compensated area appeared reduced in SM compared with CTRL when expressed as work rate differences between VT_1_ and VT_2_ (39 ± 14 vs 50 ± 13 W, respectively; *P* = 0.026; *g* = −0.81, moderate; 95% CI = −0.03–1.64). By contrast, it was similar between the two groups when expressed as V̇O_2_ differences between VT_1_ and VT_2_ (434 ± 193 vs 536 ± 137 mL·min^−1^ for SM and CTRL, respectively). Work rate corresponding to LT was lower in SM compared with CTRL (186 ± 15 vs 202 ± 26 W, respectively; *P* = 0.010; *g* = −0.98, moderate; 95% CI = 0.14–1.83) despite similar [La^−^] blood lactate concentration (3.68 ± 1.13 vs 3.36 ±1.12 mmol·L^−1^ for SM and CTRL, respectively). The [La^−^] pattern during the incremental test is shown in Figure 3.

During submaximal work rates as shown in Figures 1–3, no differences were observed in all the investigated variables, except for SV and $\dot{Q}$, which resulted different in the third and fourth work rates.

**FIGURE 4:**
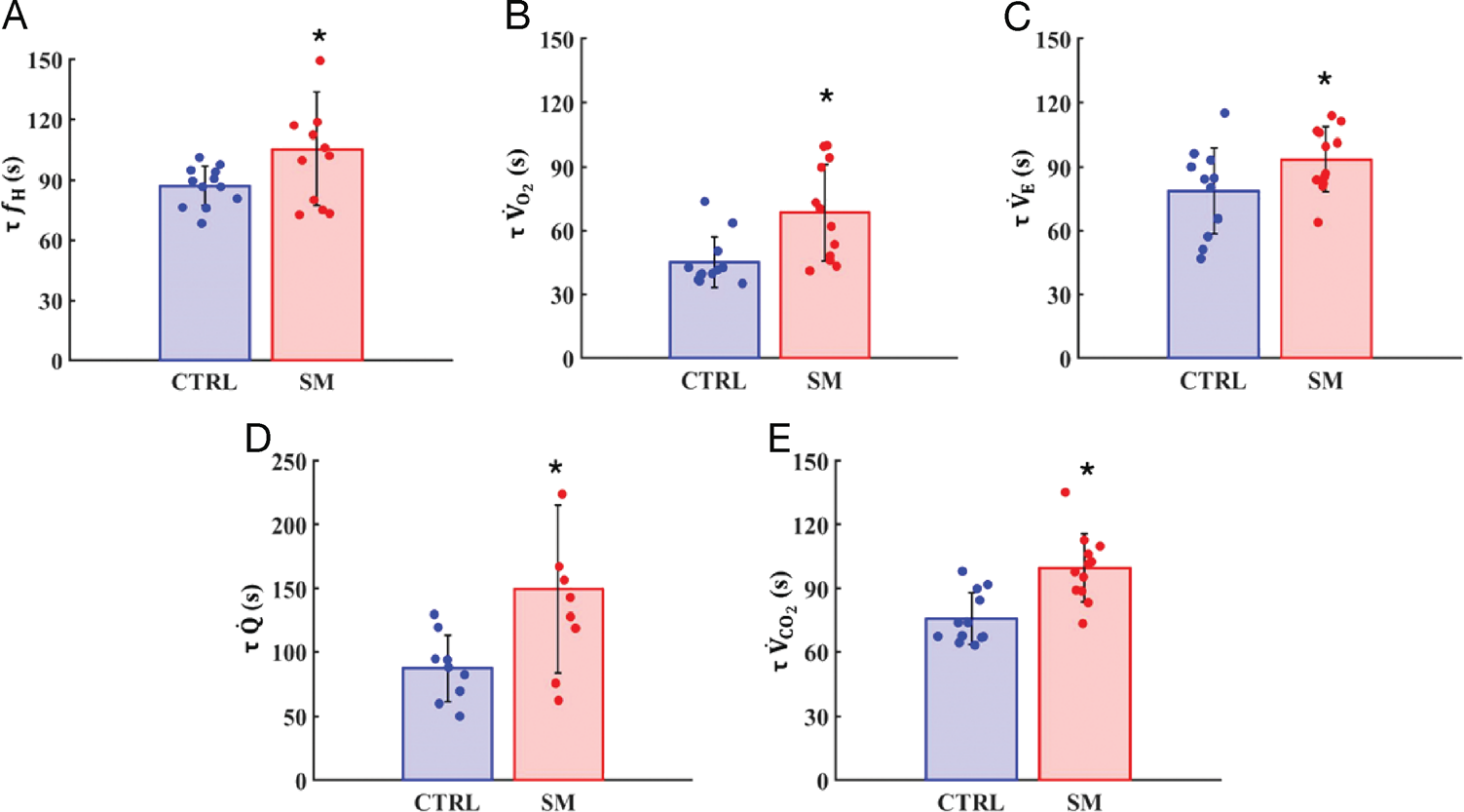
Time constant (*τ*) values of pulmonary oxygen uptake $({\dot{V}}_{O_{2}}$, A), expiratory ventilation $({\dot{V}}_{E},$ B), heart rate (*f*_H_, C), cardiac output ( $\dot{Q}$, D; CTRL = 9; SM = 9), and carbon dioxide production $({\dot{V}}_{{\mathrm{CO}}_{2}},$ E). Data are presented as mean ± SD. * *P* < 0.05 vs CTRL.

**FIGURE 5:**
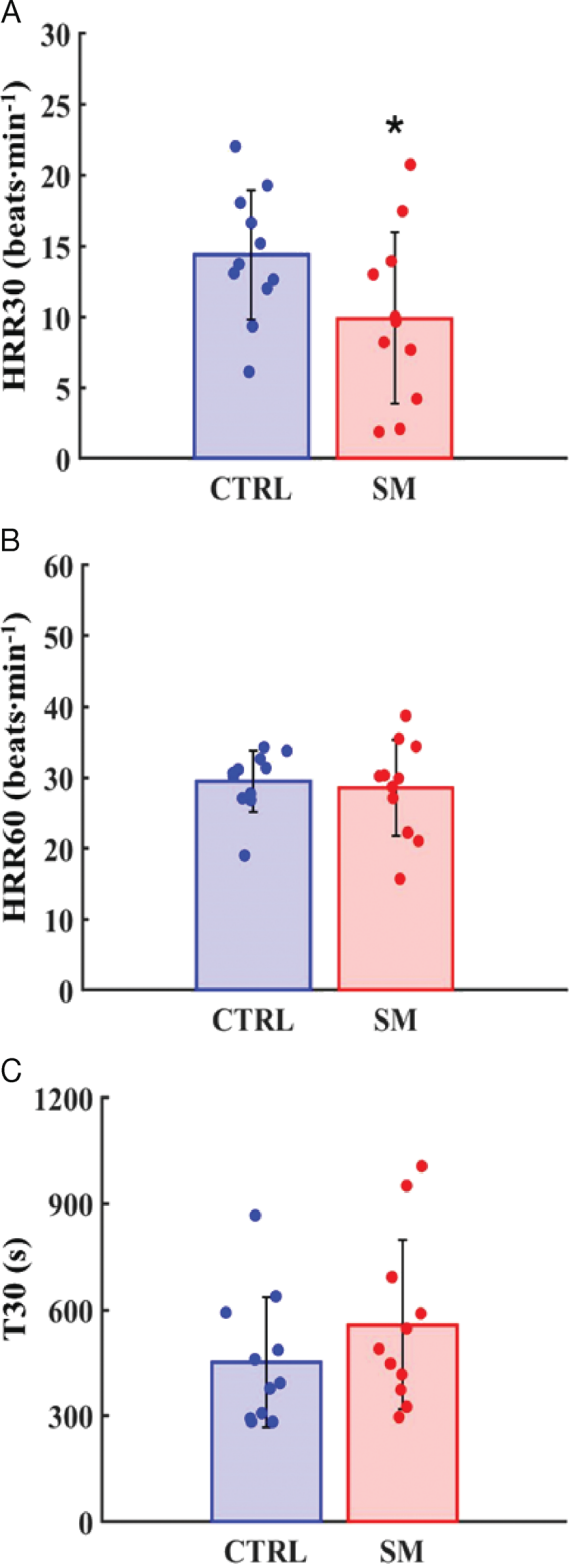
HRR30, heart rate recovery at 30 s (A); HRR60, heart rate recovery at 60 s (B); T30, negative reciprocal slope of the linear regression of *f*_H_ natural logarithm vs time in the first 30 s of recovery (C). Data are presented as mean ± SD (CTRL = 11; SM = 11). * *P* < 0.05 vs CTRL.

During recovery, SM exhibited longer *τ* compared with CTRL in *f*_H_ (+21%; *P* = 0.022; *g* = −0.84, moderate; 95% CI = 0.00–1.67; Fig. 4A), $\dot{Q}$ (+52%; *P* = 0.009; *g* = −1.18, moderate; 95% CI = 0.18–2.18; Fig. 4D), V̇O_2_ (+52%; *P* = 0.002; *g* = −1.24, large; 95% CI = 0.37–2.12; Fig. 4B), V̇_E_ (+19%; *P* = 0.027; *g* = −0.80, moderate; 95% CI = −0.03–1.63; Fig. 4C), and V̇CO_2_ (+32%; *P* < 0.001; *g* = −1.63, large; 95% CI = 0.71–2.55; Fig. 4E). In addition, as shown in Figure 5, SM were characterized by lower HRR30 compared with CTRL (−31%; *P* = 0.032; *g* = −0.80, moderate; 95% CI = −0.05–1.65; Fig. 5A) but similar HRR60 and T30 (Fig. 5B–C).

## DISCUSSION

This study represents the first endeavor to determine the effect of CS on the cardiorespiratory and metabolic response during and after a maximum incremental test in young, physically active SM without known lung or cardiovascular disease. The main findings of the present study were that SM demonstrated: (i) lower V̇O_2_ and ${\dot{V}}_{E}$ at peak incremental exercise with no differences during the test and (ii) slower cardiorespiratory and metabolic kinetics during the recovery phase, as witnessed by longer *τ* for *f*_H_, $\dot{Q}$, V̇O_2_, V̇_E_, and V̇CO_2_. These results are compatible with an early CS-related impairment of the cardiorespiratory and metabolic function even in young individuals without known lung or cardiovascular disease and with relatively short smoking history.

### Preliminary considerations

In line with previous studies involving young SM (22,23), no differences in the investigated static lung volumes were identified between the two groups (Table 2). This finding could be explained by the fact that in our physically active SM with a relatively short smoking history, the airflow restriction through bronchial remodeling that occur only upon prolonged CS exposure (16,46,47) was not probably impactful.

However, abnormalities in SM emerged in dynamic lung volumes. In particular, a reduction in PEF, indicating an increased airway resistance (36,48), and in MEF75% and FET100%, highlighting a reduction in lung elastic recoil and in the caliber of lung airways (36,48), was present. This scenario could be supported even more by a decreased MVV, which suggests a diminishing respiratory endurance, being not attributable to body size differences between the two groups (49).

### Effect of CS on cardiorespiratory and metabolic response at peak exercise

Young, physically active SM without known lung or cardiovascular disease exhibited a lower V̇O_2max_ and ${\dot{W}}_{\max}$ compared with CTRL. The few studies investigating the maximum aerobic capacity in relatively young SM yielded to questionable results because of either the lack of a CTRL group or their failure to match CTRL for exercise habits (8,12,18–20). The lower V̇O_2max_ previously observed in SM was attributed to a reduction in O_2_ carrying capacity due to a higher HbCO level. Kobayashi and colleagues (8) reported indeed a 5.5% HbCO, even if SM abstained from smoking overnight before the test. This level exceeded the 4.3% threshold established as the minimum level affecting ${\dot{V}}_{O_{2}\max}$ (50). Although HbCO concentration was not measured in the present study, we can reasonably infer a similar result in SM. Another possible explanation for the lower aerobic capacity may involve an increased skeletal muscle fatigability, as previously observed in young SM (22 ± 2 yr) with a short smoking history (2.5 ± 3.1 pack years) during a small muscle mass exercise (51). An altered fiber-type composition and oxidative capacity, reflected in lower mitochondrial volume and activity (52), could indeed cause this phenomenon. Considering that in our study SM and CTRL were matched not only for age and anthropometric characteristics but also for physical activity levels, the V̇O_2_ and ${\dot{W}}_{\max}$ reduction also suggests the predominance of the detrimental role of CS over the advantages of regular aerobic training.

Coherently with V̇O_2max_ and ${\dot{W}}_{\max}$ reduction, SM exhibited a lower V̇_E max_ mainly due to a lower *f*_R max_. An autopsy study revealed higher lung parenchyma stiffness in SM (~7 yr of smoking history) due to alterations in the structure of the collagen fibers that affect the structure and the mechanical performance of the lungs (53). Therefore, it seems reasonable to consider a reduced *f*_R max_ as an early sign of lung parenchyma stiffness.

Interestingly, the nearly identical RPE values at peak exercise seem to suggest that SM did not terminate exercise earlier than CTRL for respiratory dyspnea or muscular discomfort.

### Effect of CS on cardiorespiratory and metabolic response at submaximal exercise

At submaximal exercise, the V̇O_2_ at the same mechanical power was similar between the two groups in all the investigated workloads, indicating that CS did not alter the mechanical efficiency. This lack of difference that is in contrast with our hypothesis may be explained by the young age and the fitness level of SM, which could have counterbalanced CS detrimental effects during submaximal exercise.

Noticeably, the earlier LT observed in SM and the higher V̇CO_2_ in the last two work rates of the submaximal phase could reflect an early anaerobic metabolism utilization and, thus, the premature muscle fatigue onset. This interpretation is also supported by the muscle fiber shift from oxidative to glycolytic fiber types reported in the literature in SM (4,54,55).

A lower work rate and V̇O_2_ at both VT_1_ and VT_2_ was found in our SM. Plausible explanations encompass several factors, among which a compromised redistribution of blood flow to exercising muscles, attributed to vasoconstrictive agents present in CS and their effect on β-adrenergic responsiveness (56,57). Moreover, the leftward shift of the oxyhemoglobin dissociation curve induced by elevated HbCO concentrations may have taken place (4,58). Furthermore, CO and reactive oxygen species may lead to endothelial dysfunction, increased vascular resistance, impaired O_2_ extraction, and reduced mitochondrial oxidative capacity (17,59). Another possible explanation for VT differences might be related to abnormalities in the O_2_ transfer from the pulmonary system to arterial blood in SM. However, this aspect has been so far never investigated in young, physically active SM without known lung or cardiovascular disease and should be the object of future studies. Conversely, reduction in the $\dot{Q}$ cannot be responsible for a diminished O_2_ transport, as it is not supported by our data.

The observed higher SV and consequently $\dot{Q}$ at submaximal work rates in SM could be a compensatory mechanism at that exercise intensity for the assumed lower arteriovenous O_2_ difference (Fick’s principle) to deliver the same O_2_ flow to the mitochondria, thus counterbalancing to the reduced peripheral O_2_ extraction capacity.

SM showed a lower compensated area of the incremental test, which reflects the blood buffering capacity (41), suggesting a CS-related impairment in this function.

VT and *f*_R_ in the present study had a different pattern compared with previous studies on SM (16,46). Indeed, previous research has suggested that to overcome structural limitations induced by CS, rapid, shallow breathing can reduce respiratory muscles fatigue and maximum inspiratory muscle effort, optimizing the O_2_ cost of breathing. Specifically, for a given V̇_E_, a combination of smaller V_T_ and higher *f*_R_ is most efficient in reducing loading caused by increased elastic forces against which SM need to breath, thereby enhancing endurance of the inspiratory muscles (16,46). However, when the exercise intensity rises, this strategy becomes ineffective as dead space and the O_2_ cost of breathing increase with *f*_R_ (16,46). The absence of this behavior in our study may be due to the difference in the sample characteristics. Our study, indeed, focused on young, physically active SM, whereas these previous studies enrolled sedentary, middle age SM without COPD. These findings align with the lack of difference in ventilatory efficiency (i.e., V̇_E_/V̇_CO2_), which is still preserved.

### Effect of CS on the recovery phase

Few studies have been so far investigated the effects of CS on the cardiorespiratory and metabolic kinetics after exercise, and they have primarily focused on elderly, sedentary, and symptomatic SM, often affected by COPD. Moreover, among these studies, only one has evaluated the cardiorespiratory and metabolic kinetics after a moderate exercise, rather than maximal exercise as conducted in our study, in young sedentary SM (60).

As hypothesized, the cardiorespiratory and metabolic variables in the present study exhibited slower recovery kinetics in SM. The longer *f*_H_ and $\dot{Q}$ kinetics observed in SM of our study seem to denote that, despite their short history of CS, SM presented reduced vagal modulation capacity. Indeed, nicotine is known to induce a sympathetic overdrive that, in turn, prompts the adrenal medulla to enhance the release of both epinephrine and norepinephrine into the systemic circulation. The stimulation of catecholamine secretion, together with a decrease in the production of prostacyclins (potent vasodilators), results in an acute elevation in *f*_H_ (61). Moreover, as part of a cascade effect, there might have occurred a concurrent peripheral sympathetic excitation and an augmented sensitivity of peripheral chemoreceptors as reported in previous studies (57,62).

The CS-induced reduction in vagal cardiac modulation (56) and a shift of the sympathovagal balance toward greater sympathetic dominance (21) are evident even during the recovery phase after a maximal exercise. Specifically, SM exhibited a smaller HRR30, mainly dependent on impairment in the parasympathetic reactivation, as well as delayed *f*_H_ *τ*, also dependent on parasympathetic reactivation and the successive sympathetic tone withdrawal and the clearance of accumulated metabolites of the energy processes (i.e., lactate, H+, Pi) (63).

The delayed recovery of cardiovascular response to exercise may be reflected in slower ${\dot{V}}_{O_{2}}$ kinetics because V̇O_2_ is determined by $\dot{Q}$ and arterial–venous O_2_ difference. Noteworthy, the slower kinetics are observed alongside a lower V̇O_2_ at peak of exercise. This is particularly interesting because it indicates that SM took a longer recovery time, even when the delta required to classify as recovery is lower. Moreover, the slower V̇O_2_ kinetics during the recovery phase could be attributed to a larger O_2_ debt accumulation in SM compared with non-SM. Support of this hypothesis was found in COPD patients, who showed slower recovery time for PCr (64) as well as decreased activity of several oxidative enzymes, among which citrate synthase and phosphofructokinase (65).

Although the physiological mechanisms underlying the slower V̇_O2_ kinetics during exercise recovery are not completely understood, they appear to be related at least in part to the prolonged recovery of energy stores in the peripheral skeletal muscles (32,66,67). In principle, an increased ATP utilization or a decline in ATP production efficiency may contribute to the increased V̇O_2_ (68). Some studies conducted on COPD patients and animal models exposed to CS have shown reductions in skeletal muscle fiber size, oxidative enzyme activity, capillary regression, and a shift in muscle to glycolytic fiber types (4,54,55). The persistence of elevated O_2_ demand postexercise could be also the causes of the slower ${\dot{V}}_{E}$ kinetics.

### Study limitations

This study comes with some known limitations. It lacks data about plethysmographic lung volumes, lung diffusion, the amount of HbCO, and the determination of O_2_ extraction at the muscle level to provide a more comprehensive picture of the integrated heart–lung–muscle system. In addition, this study investigated only male participants. Moreover, during the recruitment process, a self-reported questionnaire (i.e., IPAQ) was used to match SM and CTRL for physical activity level. Although this questionnaire is validated (69), it relies on the ability of individuals to accurately report their physical habits. Lastly, in this study, only a single trial was conducted. This could represent a limit to the study as the ${\dot{V}}_{O_{2}}$ and *f*_H_ kinetics analyses are typically performed using data from repeated trials to enhance the confidence in the fitting. However, this procedure applies mostly on the on-phase transition to exercise. During recovery, the V̇O_2_ kinetics has been found to be more reproducible than during exercise on-phase, particularly when fitted with a monoexponential function (43,70,71).

## CONCLUSIONS

CS seems to have detrimental effects on cardiorespiratory and metabolic response at the peak and after an incremental exercise in young, physically active males without known lung or cardiovascular disease. In fact, despite young age and the fitness level, SM were characterized by slower cardiorespiratory and metabolic kinetics during the recovery phase. Considering that the recovery kinetics of V̇O_2_ after whole-body exercise has been used as an index of oxidative capacity, the results of our study suggest that young, healthy, and physically active SM exhibited a lower cardiorespiratory fitness and higher risk (30). Moreover, the blunted HRR suggests a link to increased cardiovascular risk in the general population that may also be applied to young, physically active SM without known lung or cardiovascular disease.

Practically, this study is expected to increase awareness regarding the detrimental consequences of CS, empowering individuals to make well-informed choices about their health, with a particular focus on the younger population, considering that the impaired cardiac response during the recovery phase estimates a major overall cardiovascular risk (26,27,29).
